# Challenges in blood pressure measurement in children with obesity: focus on the cuff

**DOI:** 10.1007/s00467-025-06678-5

**Published:** 2025-02-05

**Authors:** Kleo Evripidou, Athanasia Chainoglou, Vasilios Kotsis, Stella Stabouli

**Affiliations:** 1 1st Department of Pediatrics, Hippokration General Hospital, Aristotle University Thessaloniki, Thessaloniki, Greece; 2 3rd Department of Internal Medicine, Papageorgiou Hospital, Aristotle University Thessaloniki, Thessaloniki, Greece

**Keywords:** Obesity, Children, Adolescents, Blood pressure, Hypertension, Cuff, Conical

## Abstract

Childhood obesity is a well-known risk factor for primary hypertension (HTN), especially during puberty. Validated automated oscillometric devices based on universal protocols using proper cuffs according to arm sizes must be selected for blood pressure (BP) measurement in clinical practice. Recent recommendations have addressed the importance of accurate BP measurement in patients with obesity. The American Heart Association (AHA) suggests using a cone-shaped cuff, while the European Society of Hypertension (ESH) emphasizes the significance of cuff characteristics based on arm size. The applicability of cone-shaped cuffs over cylindrical cuffs has been studied in adults with obesity. Most studies focused on the upper arm’s shape and provided evidence that the use of cylindrical cuffs may overestimate BP in individuals with obesity, while the conical cuff was proposed as a proper alternative. However, current validation studies for BP measurement devices have not included the arm’s size as a recruiting criterion, which may compromise the accuracy of devices in individuals with obesity. This review discusses the role of arm characteristics in cuff selection and the potential role of conical cuffs as an alternative to commonly used cylindrical ones in BP measurement in pediatric patients with obesity.

**Graphical abstract:**

**Figure:**
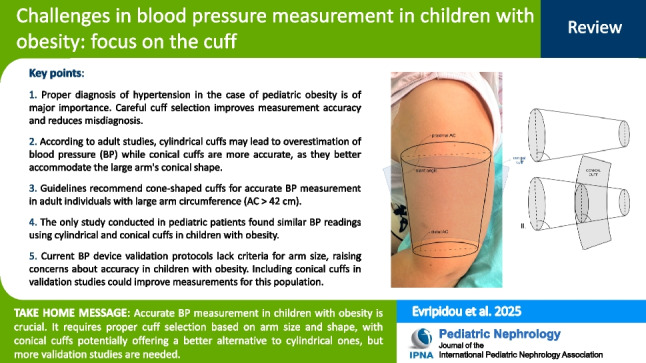
A higher resolution version of the Graphical abstract is available as Supplementary information

**Supplementary Information:**

The online version contains supplementary material available at 10.1007/s00467-025-06678-5.

## Introduction

Excess weight gain and visceral obesity are well-known causes of hypertension (HTN) [1]. Increased adiposity has been shown to relate to increased HTN rates, and body mass index (BMI) was closely associated with systolic blood pressure (SBP) elevation [2–4]. Approximately 30% of the pediatric population with obesity has elevated blood pressure (BP) or ΗΤΝ [5]. Serial cross-sectional analysis in the NHANES (National Health and Nutrition Examination Surveys) [6, 7] studying BP levels in large cohorts of children and adolescents found a higher risk of HTN in children with obesity compared to those with normal weight. As BMI increases, there is an evidenced risk for higher BP values [8–10], while for BMI ≥ 95th percentile, the prevalence of HTN increases to 11% [11]. Similar associations were found between increasing waist and hip circumference and high BP [7, 9]. Children with obesity have significantly higher ambulatory blood pressure (ABP), specifically higher mean 24-h, daytime, and nighttime SBP [12]. Inversely, when excess body weight is reduced, SBP may decrease [13, 14].

Considering the complications of obesity itself during childhood [15, 16] and the increased risk of HTN in adulthood [17, 18], it becomes obvious that HTN in obese children and adolescents represents a major health problem in children that should not be misdiagnosed. Currently, office BP is the recommended method for the diagnosis and screening of HTN in the pediatric population. Precise BP readings in appropriate settings are crucial for pediatric patients to avoid misdiagnosis, while the process of BP measurement could be more challenging compared to adults, especially in younger children [19]. The definition of BP status in children is based on age, sex, and height percentile using the 2016 European Society of Hypertension (ESH) or the 2017 American Academy of Pediatrics (AAP), as the most recent guidelines [20, 21]. For adolescents older than 16 years of age according to the ESH guidelines or for adolescents older than 13 years according to the AAP 2017 guidelines, relevant adult thresholds are recommended for the diagnosis of HTN. The diagnosis of HTN requires three different visits with standardized measurements using either auscultatory or validated oscillometric devices [21]. As reference values are established from auscultatory data, challenges remain in office BP measurement interpretation with oscillometric devices. Using a validated oscillometric device increases the reliability of BP measurements, but measurements usually vary depending on the device used, while the availability of validated BP devices for children is limited [22].

To ensure BP measurement devices’ reliability up to the extreme of the declared arm size range, a balanced distribution of arm circumferences (ACs) should be taken into account when selecting the right cuff. In general, when using too short or too narrow cuffs, the likelihood of ‘undercuffing’ increases, and as a result, it leads to inaccurate BP measurements, causing an overestimation of the true BP, while on the other side, ‘overcuffing’ using too wide or too long bladders leads to BP underestimation [23, 24]. Selecting the proper cuff in people with obesity could be determined by the arm’s size and shape. The amount of adipose tissue and the conical shape of the arm, along with the large AC and the disproportionate length, are usual arm characteristics observed in individuals with obesity [25]. These individual characteristics may compromise the accuracy of BP measurements despite using the proper cylindrical cuff size due to improper cuff fit on the arm. This review discusses the role of arm characteristics in cuff selection and the role, if any, of conical cuffs as an alternative to commonly used cylindrical ones in BP measurement in pediatric patients.

## Mechanical insights of oscillometric BP measurement

The compression of the brachial artery caused by the cuff bladder is a key parameter in analyzing BP readings. The amount of the applied pressure that causes alterations in the artery’s volume is determined by how the cuff wraps around the arm while ensuring its uniform distribution to the tissues below the cuff. It has been suggested that the pressure decreases by approximately 30% at the cuff’s edge, even though the pressure from the bladder remains constant across the entire upper arm, while the transmission ratio should be 1:1 within the area covered by the cuff. Changes in elasticity have a small impact on this transmission, but the excess soft tissue may lead to limited compressibility and reduced signal transmission, resulting in erroneous measurements [26]. The pressure decrease has been reported to be more significant when a cylindrical cuff was attached to a tronco-conical large arm due to an air gap between the arm’s surface and the cuff above the elbow. This pressure drop was directly related to the inflating pressure, suggesting that in individuals with conical-shaped arms, cylindrical cuffs could result in less accurate BP measurement [27]. The cuff’s function differs between auscultatory and oscillometric BP techniques, as in traditional auscultation, it applies specific pressure to the artery, while in oscillometry, the cuff acts as a signal sensor [23]. Varying stiffness levels on the artery’s wall do not impact the precision of BP readings taken through oscillometry, which seems to be a benefit of this technique [26].

A significant influence of arm fat index (AFI) on BP readings in healthy young adults was demonstrated using machine learning models. There was a negative correlation between SBP and AFI but a positive correlation between SBP and AC, supporting the evidence for the effect of arm characteristics on accurate BP measurement [28]. The subcutaneous fat layer was suggested to play an important role in the transmission of pressure during the inflation of the cuff. The required pressure to occlude the artery increases according to the fat mass [29].

## Challenges in cuff selection in individuals with obesity

The characteristics of the optimal cuff in selected populations, such as individuals with obesity, have been a topic of extensive discussion since the twentieth century [30]. Inappropriate cuffs can lead to misclassification of BP in patients with obesity [31]. The arm shape in these individuals is often tronco-conical, varying by sex, BMI, and AC [32–34]. As the arm size increases, variations in compression of the brachial artery on the arm’s surface can occur [35]. Thus, BP may be overestimated because the cuff’s lower part remains loose above the elbow and expands unevenly while inflating, resulting in reduced pressure on the brachial artery beneath this arm section [23].

Cylindrical cuffs are less likely to properly fit and cover the required arm’s surface to get precise measurements and diagnosis for HTN [27, 36, 37]. Prior research in adults found that as upper AC and BP levels increase, the accuracy of BP measurements using conical cuffs compared to cylindrical ones increases [27, 36]. Excessive subcutaneous fat decreases the pressure transmission, resulting in higher BP readings using cylindrical cuffs [38]. Moreover, conical cuffs require lower pressure to inflate than cylindrical ones [26].

Wide-range cuffs may be suitable for patients with obesity, featuring smaller bladders and software that adjusts monitor parameters to the arm characteristics [39]. For ACs of 35–45 cm and 45–52 cm, the American Heart Association (AHA) suggests bladder lengths of 16 cm and 20 cm [40]. In cases of short upper arm length, 16-cm bladders are preferable, as larger cuffs may not fit properly. Of note, the current range of brachial conical cuffs available in the market is limited. Two conical adult cuffs (44–66 cm by W. A. Baum Co., and 22–42 cm, M–L by Microlife Corporation) and a validated BP monitor for bariatric patients (UA-789-XL AC, LifeSource, Inc.) with a cone-shaped cuff are only available.

Evidence for the importance of a correctly fitting upper arm cuff for the diagnosis of HTN in patients with obesity was supported in a diagnostic accuracy review study. BP measurements with an appropriately fitted cuff in adults with large AC demonstrated adequate sensitivity and specificity in diagnosing HTN compared to the index cuff or the invasive BP measurement method [41]. Wrist or forearm BP measurements could be used as an alternative due to severe obesity when technical difficulties occur [41–43]. A forearm conical cuff (General Electric, GE, Healthcare) in adults with obesity was the first attempt to validate a cuff for noninvasive radial artery BP measurements from the forearm [44, 45]. Comparison of different cuff locations or wrapping techniques (including conical wrapping) with invasive intra-arterial BP measurement as the gold standard proved limited precision [42, 46, 47]. Although devices for BP measurement in alternative positions to the upper arm are available, proper validation for clinical use in obesity remains undeveloped.

## Conical cuffs

### Findings in adults

**Fig. 1:**
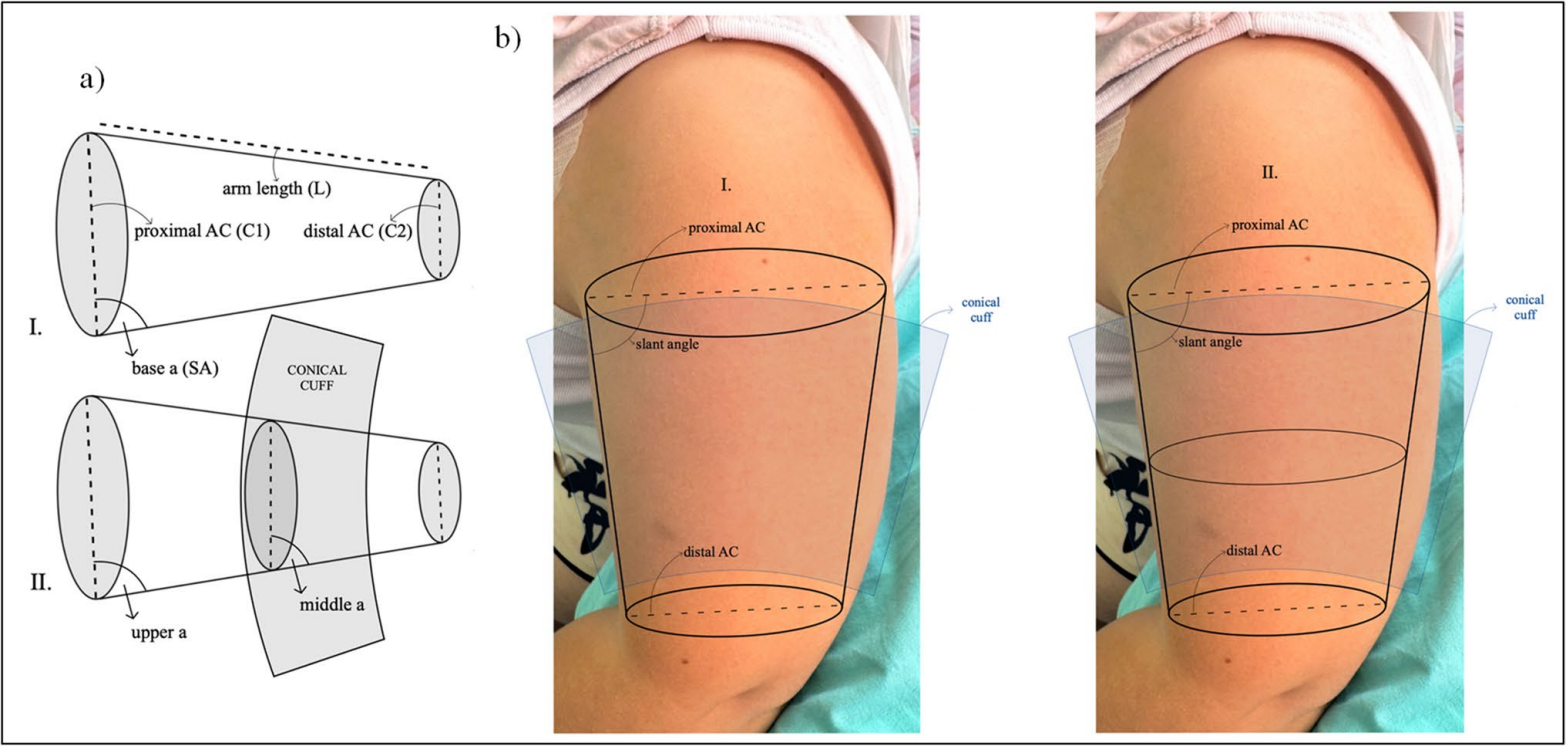
Representation of the upper arm models and the conical cuff in large-arm subjects. The upper arm can be modeled as a single truncated cone or two. In typical arms, the slant angles of the cones are similar, but in larger arms, the middle slant angle is smaller than the upper one, so the use of a conical cuff can be considered [37]. **a)** I. One truncated-cone model, II. two truncated-cones model; base a = slant angle (SA) = upper a—the angle concerned with the slant surface and the proximal arm base-arm circumference (AC) (C1) below the axilla, middle a = the angle concerned with the slant surface and the distal arm base-AC (C2) above the antecubital fossa, SA = arccosine [(C_1_ − C_2_)/(2π × L)] × (360/2π) [37]. **b)** Example of a pediatric patient (AC = 35 cm) with a large arm and the models applied. I. One truncated-cone model, II. two truncated-cone

Cone-shaped cuffs have been studied for their performance over the commonly used cylindrical (or rectangular) cuffs to assess BP levels in adults with obesity [24, 27, 32, 35–37, 41, 48, 49]. Most studies focused on studying the shape of the upper arm and especially the slant angle (the angle concerned with the slant surface and the proximal base of the cone) due to the conical shape of large arms with excessive middle AC. The calculation of the angle size in degrees is based on the following equation:

$$Slant\;angle\;\left(SA\right)=arccosine\left(\frac{C1 - C2}{2\pi \times L}\right)\times \left(\frac{360}{2\pi }\right)$$

in which ‘C_1_’ is the proximal AC, ‘C_2_’ is the distal AC, and ‘L’ is the arm length [27, 34, 35]. The two truncated cone models with bases in the proximal and the distal ACs have also been proposed as a representation of the upper arm shape [23, 35–37]. The difference between the slant angle of the upper cone (upper a) and the slant angle of the lower cone (middle a) determines whether a single or two truncated cones approximate the arm (Fig. 1). As the arm’s size increases, the difference between the angles of the two truncated cones increases correspondingly. In some individuals with obesity, the shape of the upper arm is not a simple tronco-conical structure but is better described as the combination of two truncated cones with differing slant angles. A positive difference between the angles occurs when the lower half of the arm is more conical. In this case, the approach of two truncated cones in ACs greater than 33 cm is suggested to improve accuracy [37].

The value of 85° for SA has been proposed for the upper limit to use a conical cuff in adults [34, 36, 49] as the mean SA of the truncated cone presenting the upper arm was found to be 85±1.4° [27]. The SA decreases as AC increases. The difference between distal and proximal AC represents the difference between the shape of the upper and lower half of the arm and the two truncated cones, which cannot always be eliminated with the use of conical cuffs [35, 37]. Conical cuffs of different sizes would be useful for increasing ACs; the conical shape of the arm is also progressively increasing in size [25].

**Fig. 2:**
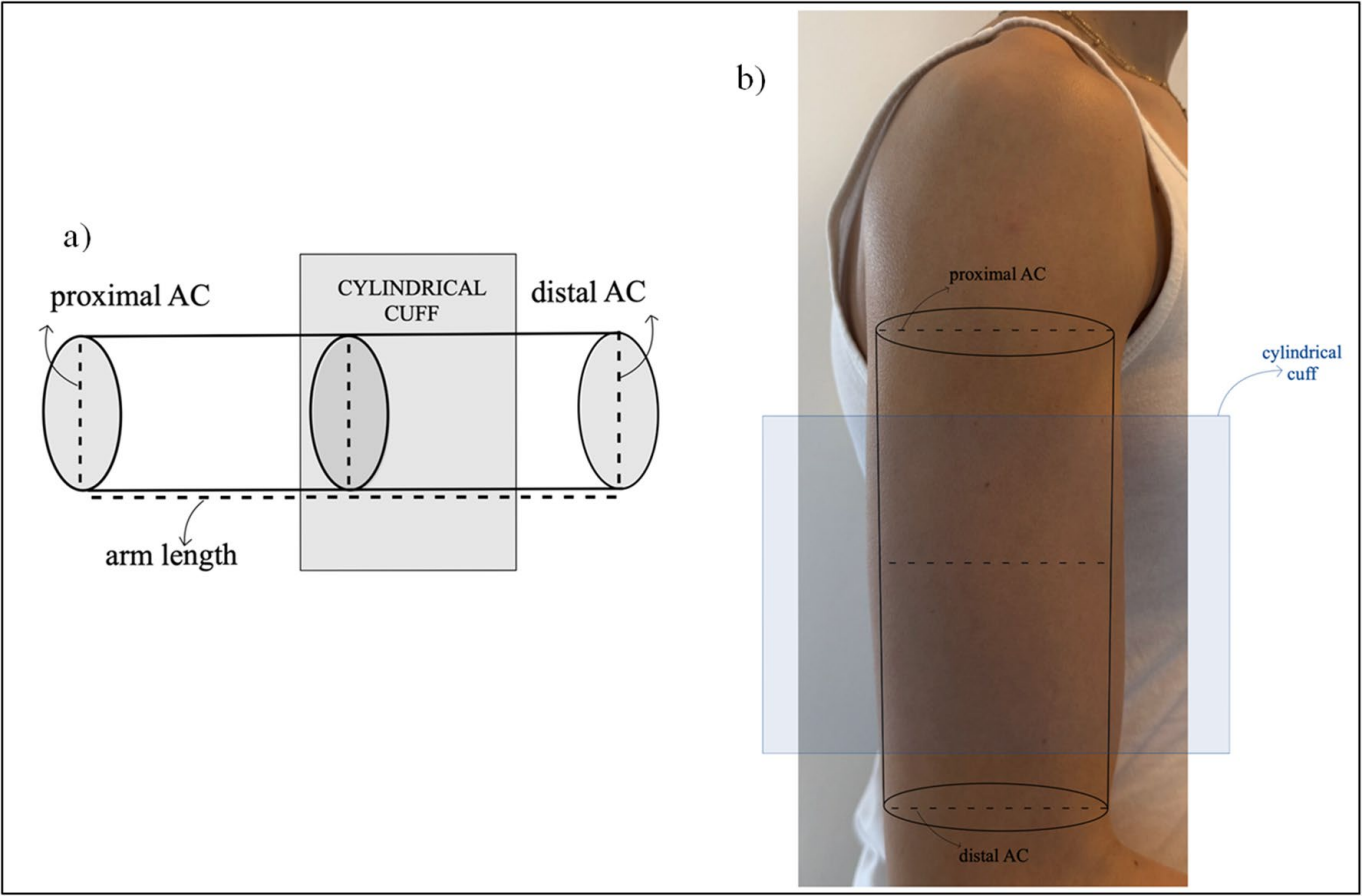
Representation of the upper arm model and the cylindrical cuff in typical arm subjects. **a)** Cylindrical arm model, proximal arm circumference (AC) of the arm base below the axilla = distal AC of the arm base above the antecubital fossa. **b)** Example of a pediatric patient (AC = 24 cm) with a typical arm and the cylindrical model applied

The first adult study investigating the comparison between the two shapes [24] showed that cylindrical cuffs as used in typical arms (Fig. 2) may overestimate BP in individuals with large arms (AC > 34 cm) in clinical practice and proposed the use of a conical cuff [50, 51] with a slant angle of 86° that was previously designed for intra-arterial comparisons [52]. A similar study, including only individuals with an AC of 37.5–42.5 cm, found that a significant proportion of participants who were normotensive with the conical cuff were found to be hypertensive with the cylindrical one with differences reaching up to 10 mmHg for SBP [32]. In individuals with AC > 42 cm, overestimation with a cylindrical cuff was more likely to be observed in patients with high BP and larger AC, underscoring the risk of overtreating them [27]. These results were confirmed in a recent study by the same investigators comparing different sizes of conical and cylindrical cuffs in adults separated into three groups based on their AC (≤ 35 cm, 36–42 cm, and > 42 cm). Overestimation of BP was found to be significant in the group of patients with obesity and AC > 42 cm [49]. A subsequent study including only patients with obesity and AC > 40 cm supported the use of arm conicity on the selection of proper cuff reflecting its actual shape in individuals with obesity [37]. In most of the abovementioned studies, BP was measured with the auscultatory method [24, 27, 35, 37]. Finally, a single rigid conical cuff in adults was demonstrated to provide accurate measurements in patients with AC ranging from 22 to 44 cm, and AC was the only characteristic of the arm associated with the conical shape in a multiple linear analysis model [33].

### Findings in children and adolescents

Currently, the available conical cuffs are designed for adults and may not be appropriate for pediatric arms due to differences in pathophysiology and arterial properties. Aging’s effect on the mechanical properties of adipose tissue, leading to varying amounts of adiposity across ages [53, 54], along with the reduced arterial stiffness due to young age [55, 56], may affect the performance of conical cuffs in pediatric patients. Previous research in adults has provided evidence for the effectiveness of conical cuffs used with auscultatory devices [27, 32, 34, 35, 37]. In oscillometry, the cuff pressure reflects the total volume change in the artery beneath the cuff rather than just its central region, and the accuracy of BP measurement is not affected by differences in arterial stiffness [26, 32]. These can be considered as benefits of the technique, and the overestimation of BP using cylindrical cuffs should also be tested in these settings. Another aspect to consider before implementing conical cuffs in pediatric populations is the tolerability of the different cuffs, as the BP measurement requires the patient to be comfortable during the process.

We recently performed an observational study [57] including 37 patients with obesity, aged 6–16 years, to compare office BP assessed by conical and cylindrical cuffs. A validated oscillometric device was used (OMRON HBP-1320, HBP-1320-E) with both cylindrical and conical cuffs of different sizes. We found similar BP levels measured by using cylindrical compared to conical cuffs for SBP and DBP both in mean levels and z-score values. The prevalence of BP measurement in the hypertensive range also did not differ between the cuffs. Only a few individuals were found to be hypertensive with the cylindrical but normotensive with the conical cuff, suggesting that BP was not generally overestimated with the regular cuff. The discrepancy between this study’s findings and existing adult-focused literature stems from the unique anatomical arm characteristics of children and adolescents. Differences in adiposity tissues and AC due to age group could suggest that BMI may not be the ideal appropriate marker for defining the pediatric population with obesity in whom a conical cuff should be applied. This study is the only study conducted in the pediatric population, while conical cuffs should be tested in different settings and larger sample sizes for more definitive conclusions on their utility in BP measurement in children and adolescents with obesity.

## Recommendations for cuff selection in patients with obesity

Scientific boards have recently provided recommendations on the cuff’s shape for BP measurements. AHA recommends using cone-shaped cuffs with appropriately sized bladders to enhance the accuracy of BP estimation. ESH also discusses the importance of cuff properties based on arm characteristics [48, 58, 59]. The conical cuffs with an 85° SA are recommended in case of AC > 42 cm to ensure accurate BP measurement by proper compression of the upper arm. The bladder’s dimensions according to AC, SA, and materials are further described. Wide-range cylindrical cuffs coupled with validated oscillometric devices using an appropriate software algorithm are also suggested for use in individuals with obesity. Other alternatives, such as wrist or forearm devices, could be considered for arms with short lengths, although their accuracy is debatable [48].

## Validation issues of BP measurement devices in individuals with obesity

All automated oscillometric devices used either for office BP measurement, home BP monitoring, or ABP monitoring (ABPM) should be validated based on universal protocols using preferably the auscultatory method as a reference standard. According to AAMI/ESH/ISO recommendations, BP measurement devices intended for use in the general population, both for adults and children, should include at least 35 subjects aged 3–12 years and at least 50 subjects > 12 years, separately reporting SBP and DBP differences in those groups. Korotkoff sounds K1 and K5 of auscultation are used for reference systolic and diastolic BP, respectively [60].

In general, the established validation protocols define the sample size, the measurement process, and the required statistical analysis. The cuffs used for reference BP measurements should be described in detail (manufacturer, materials, bladder characteristics). The bladder’s length should cover 75–100%, and the width should cover 35–50% of the AC. For devices with multiple (*n*) cuffs, cuff-size stratified subgroups are recommended with a pre-defined minimum number of participants to be tested per cuff without the requirement of subgroup analysis. Each cuff should be tested on at least 1/(2 × *Ν*) of the participants, where ≥ 40% of participants should have mid AC within the upper and the lower half of the specified range of use for each cuff [60].

Although validation protocols describe the percentage of the AC that should be encircled by the cuff, they do not refer to the patients’ arm size. This can be considered a major limitation of the current validation recommendations. As arm size, expressed as the mid AC, is not included in the criteria for population recruitment, the accuracy of the tested devices can be considered debatable in individuals with obesity and large arms. This issue also affects validated BP measurement devices in children and adolescents with obesity.

In a recent ESH position paper, it has been proposed that conical cuffs with proper dimensions and fit should also be used coupled with the reference auscultatory BP devices in validation studies, including individuals with obesity [48]. As validation protocols do not refer to this population specifically, none of the available validation studies for BP measurement devices used in clinical pediatric practice was carried out enrolling patients with obesity.

## Conclusions

Children and adolescents with obesity represent a special population at risk of HTN in whom a primary goal is to ensure the integrity of BP values, avoiding misdiagnosis and under- or overtreatment. The individual anthropometric characteristics, including arm shape, may pose further significant challenges to BP measurement that are currently under-recognized in clinical practice. There is a lack of reliable reference measurements for cuff validation studies in populations with large arms, and technical challenges persist in this group underlying the need for cuffs and possibly devices specifically developed and tested in patients with obesity. The size, shape, and slant angle of conical cuffs, along with the materials used and properties of the inflatable bladder, are all factors that affect BP measurement. However, only one study has examined the use of conical cuffs in children and adolescents with obesity, showing no significant differences in office BP levels between the two shapes of cuffs [57]. The optimal characteristics of the cuff selection for accurate BP measurement remain a topic for discussion and further research. While conical cuffs could be considered an option for office BP readings in adults with obesity, their reliability needs to be specifically evaluated in pediatric clinical practice settings.

## Supplementary Information

Below is the link to the electronic supplementary material.

Graphical abstract (PPTX 2.07 MB)

## Data Availability

Data sharing does not apply to this article as no datasets were generated or analyzed during the current study.
