# Supplementary material for: Challenges in blood pressure measurement in children with obesity: focus on the cuff
Source: Pediatr Nephrol. 2025 Feb 5;40(11):3359–66. doi: 10.1007/s00467-025-06678-5 (PMC12484095; doi:10.1007/s00467-025-06678-5)
Supplement: Supplementary file 1 — Graphical abstract (PPTX 2.07 MB) [file 467_2025_6678_MOESM1_ESM.pptx]

## Slide 1
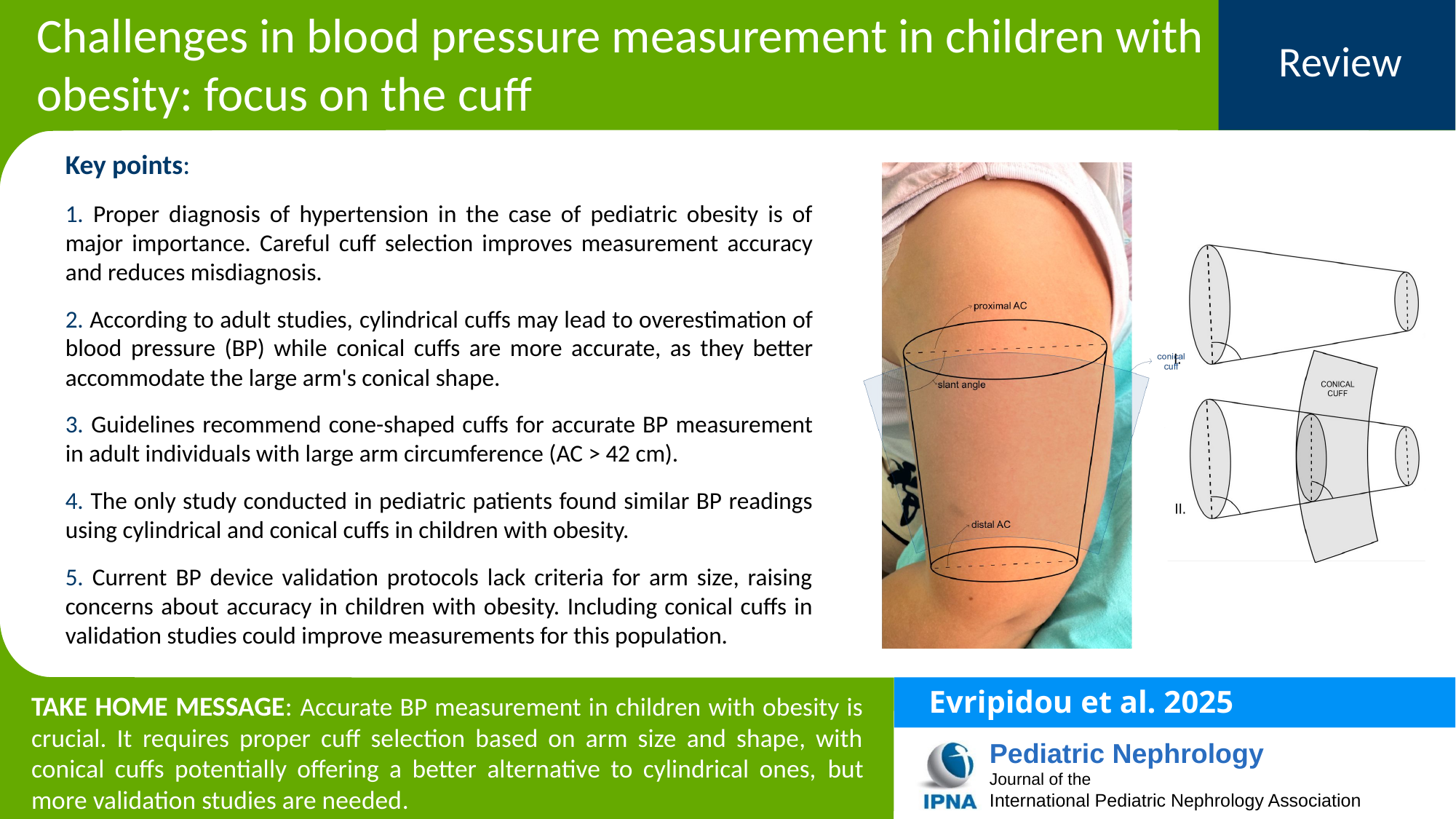

Challenges in blood pressure measurement in children with obesity: focus on the cuff
Key points:
1. Proper diagnosis of hypertension in the case of pediatric obesity is of major importance. Careful cuff selection improves measurement accuracy and reduces misdiagnosis.
2. According to adult studies, cylindrical cuffs may lead to overestimation of blood pressure (BP) while conical cuffs are more accurate, as they better accommodate the large arm's conical shape.
3. Guidelines recommend cone-shaped cuffs for accurate BP measurement in adult individuals with large arm circumference (AC > 42 cm).
4. The only study conducted in pediatric patients found similar BP readings using cylindrical and conical cuffs in children with obesity.
5. Current BP device validation protocols lack criteria for arm size, raising concerns about accuracy in children with obesity. Including conical cuffs in validation studies could improve measurements for this population.
Evripidou et al. 2025
TAKE HOME MESSAGE: Accurate BP measurement in children with obesity is crucial. It requires proper cuff selection based on arm size and shape, with conical cuffs potentially offering a better alternative to cylindrical ones, but more validation studies are needed.
